# Non-proteinogenic amino acids mitigate oxidative stress and enhance the resistance of common bean plants against *Sclerotinia sclerotiorum*

**DOI:** 10.3389/fpls.2024.1385785

**Published:** 2024-04-22

**Authors:** Yasser Nehela, Yasser S. A. Mazrou, Nehad A. El_Gammal, Osama Atallah, Tran Dang Xuan, Abdelnaser A. Elzaawely, Hassan M. El-Zahaby, Abdelrazek S. Abdelrhim, Said I. Behiry, Emad M. Hafez, Abeer H. Makhlouf, Warda A. M. Hussain

**Affiliations:** ^1^ Department of Agricultural Botany, Faculty of Agriculture, Tanta University, Tanta, Egypt; ^2^ Business Administration Department, Community College, King Khalid University, Abha, Saudi Arabia; ^3^ Plant Pathology Research Institute, Agricultural Research Center, Giza, Egypt; ^4^ Department of Plant Pathology, Faculty of Agriculture, Zagazig University, Zagazig, Egypt; ^5^ Transdisciplinary Science and Engineering Program, Graduate School of Advanced Science and Engineering, Hiroshima University, Higashi-Hiroshima, Japan; ^6^ Center for the Planetary Health and Innovation Science (PHIS), The International Development and Cooperation (IDEC) Institute, Hiroshima University, Higashi-Hiroshima, Japan; ^7^ Department of Plant Pathology, Faculty of Agriculture, Minia University, Minia, Egypt; ^8^ Agricultural Botany Department, Faculty of Agriculture (Saba Basha), Alexandria University, Alexandria, Egypt; ^9^ Department of Agronomy, Faculty of Agriculture, Kafrelsheikh University, Kafr El-Sheikh, Egypt; ^10^ Department of Agricultural Botany, Faculty of Agriculture, Minufiya University, Shibin El-Kom, Egypt

**Keywords:** non-proteinogenic amino acids, γ-aminobutyric acid (GABA), ß-alanine, white mold, common bean, antioxidant, *Sclerotinia*, *Phaseolus vulgaris*

## Abstract

White mold, caused by the necrotrophic fungus *Sclerotinia sclerotiorum*, is a challenging disease to common bean cultivation worldwide. In the current study, two non-proteinogenic amino acids (NPAAs), *γ*-aminobutyric acid (GABA) and *ß*-alanine, were suggested as innovative environmentally acceptable alternatives for more sustainable management of white mold disease. *In vitro*, GABA and *ß*-alanine individually demonstrated potent dose-dependent fungistatic activity and effectively impeded the radial growth and development of *S. sclerotiorum* mycelium. Moreover, the application of GABA or ß-alanine as a seed treatment followed by three root drench applications efficiently decreased the disease severity, stimulated plant growth, and boosted the content of photosynthetic pigments of treated *S. sclerotiorum*-infected plants. Furthermore, although higher levels of hydrogen peroxide (H_2_O_2_), superoxide anion (O_2_^•−^), and malondialdehyde (MDA) indicated that *S. sclerotiorum* infection had markedly triggered oxidative stress in infected bean plants, the exogenous application of both NPAAs significantly reduced the levels of the three studied oxidative stress indicators. Additionally, the application of GABA and *ß*-alanine increased the levels of both non-enzymatic (total soluble phenolics and flavonoids), as well as enzymatic (catalase [CAT], peroxidases [POX], and polyphenol oxidase [PPO]) antioxidants in the leaves of *S. sclerotiorum*-infected plants and improved their scavenging activity and antioxidant efficiency. Applications of GABA and *ß*-alanine also raised the proline and total amino acid content of infected bean plants. Lastly, the application of both NPAAs upregulated the three antioxidant-related genes *PvCAT1*, *PvCuZnSOD1*, and *PvGR*. Collectively, the fungistatic activity of NPAAs, coupled with their ability to alleviate oxidative stress, enhance antioxidant defenses, and stimulate plant growth, establishes them as promising eco-friendly alternatives for white mold disease management for sustainable bean production.

## 1 Introduction

Common bean (*Phaseolus vulgaris*; Family Fabaceae) is a legume widely cultivated for its edible seeds and seedpods. It is second to soybean in economic and societal importance as a leguminous food crop that has approximately 85% share in worldwide bean production (Amani Machiani et al., 2019). The global annual yield of common beans is over 27 million tons harvested from about 29 million ha worldwide (Gepts et al., 2008). The common bean is rich in protein and provides moderate amounts of iron, thiamin, and riboflavin to more than 300 million people worldwide (FAOSTAT, 2023; Nasar et al., 2023). There are numerous varieties of common beans, including many popular garden types such as pole, snap, string, and bush beans. Some varieties of the common bean are grown only for dry seeds, some only for the edible immature pods, and others for the seeds, either immature or mature. However, most, if not all, of these varieties are susceptible to several fungal, bacterial, and viral diseases that cause severe losses (between 20–100%) in yield and quality of common beans worldwide (Singh and Schwartz, 2010). Although angular leaf spot, anthracnose, root rots, and rust are the most severe fungal diseases, white rot disease is one of the most commonly reported diseases worldwide (Singh and Schwartz, 2010).

White mold disease of common bean is a destructive fungal disease caused by the soil-borne ascomycetous fungus *Sclerotinia sclerotiorum* (Lib.) de Bary, which can infect all above-ground parts of the plant (Schwartz et al., 2005). The disease is polyphagous and has a wide host range where it can infect approximately 400 species belonging to more than 200 genera of land plants including, but not limited to, carrot, cucumber, lettuce, onion, sunflower, and tomato (Schwartz et al., 2005; Figueirêdo et al., 2010; Elsheshtawi et al., 2017). The fungus infects old and dying flowers, then grows into neighboring tissues and organs, which are rapidly killed. Infected flowers falling onto leaves and branches will also spread the disease within a crop (Schwartz et al., 2005). The symptoms of white mold disease include a watery-brown soft rot that develops on stems, leaves, flowers, or pods, followed by a white-cottony mold. Stem infections near the soil level can lead to plant collapse (Schwartz et al., 2005). The most characteristic sign of *S. sclerotiorum* is its ability to produce black, seed-like resting structures known as “sclerotia” that might develop in the white fuzzy growths of mycelium or the stem pith of infected plants (Schwartz et al., 2005; Figueirêdo et al., 2010; Elsheshtawi et al., 2017). It is worth mentioning that sclerotia can spread from field to field through futile sanitation practices such as soil movement and/or contaminated water which makes it very difficult to completely eradicate the pathogen from infected areas (Schwartz et al., 2005).

Due to the lack of adequate host genetic resistance, the wide host range of the pathogen, and the tolerant resting bodies, management of white mold disease is extremely challenging and involves integrated multi-pronged strategies using a combination of cultural practices (Ali and Atallah, 2020), biological control (Ali and Atallah, 2020; Atallah and Yassin, 2020; Atallah et al., 2022), and chemical fungicides (O’Sullivan et al., 2021). Although the utilization of synthetic fungicides is the most effective available means against white mold disease, the frequent and extensive use of these fungicides drives major problematic traits to the environment and has undesirable impacts on humans, animals, and non-targeted microorganisms (Zubrod et al., 2019). Accordingly, intensive research to find cheap, eco-friendly alternatives that are safer for humans, animals, and the environment is a necessity. Exogenous application of non-proteinogenic amino acids (NPAAs) might be a promising potential alternative against soil-borne phytopathogens (Ren et al., 2022) to reduce the use of hazardous chemical fungicides entirely or partially.

In addition to 20 universal proteinogenic AAs, plants synthesize over 250 NPAAs that do not contribute to protein synthesis but play important roles in various aspects of plant biology. For instance, NPAAs contribute to the synthesis of compounds that are anti-herbivory, anti-microbial, response to abiotic stresses, nitrogen storage, toxins against both vertebrates/invertebrates and phytohormones (Parthasarathy et al., 2019; Trovato et al., 2021). NPAAs have been shown to have diverse functions and to be involved in plant growth, development, stress response, and metabolism (Trovato et al., 2021). NPAAs can act as signaling molecules that regulate plant growth and development by integrating the metabolic status of the plant with environmental signals, especially under stressful conditions (Vranova et al., 2011; Trovato et al., 2021). They can also serve as precursors for the biosynthesis of primary and secondary metabolites, which are essential for plant growth and defense against biotic and abiotic stresses (Vranova et al., 2011). NPAAs include but are not limited to, 2-aminoisobutyric acid, 4-aminobenzoic acid, aminolevulinic acid, *S*-aminoethyl-L-cysteine, and γ-aminobutyric acid (GABA), and *ß*-alanine.

GABA and *ß*-alanine are NPAAs that occur naturally in plants, animals, and microorganisms (Kinnersley and Turano, 2000; Parthasarathy et al., 2019). They are involved in various metabolic processes and have been shown to play multiple functions in plant responses and defense mechanisms against both biotic and abiotic stress (Parthasarathy et al., 2019; Ramos-Ruiz et al., 2019). For instance, GABA is a key metabolite for primary and secondary pathways, serving as an important intermediate of nitrogen metabolism and amino acid biosynthesis (Häusler et al., 2014; Ramos-Ruiz et al., 2019). Additionally, the GABA metabolism through the GABA shunt provides a source of carbon skeletons and energy for downstream biosynthetic pathways. For example, it enables the non-cyclic flux toward succinate outside the tricarboxylic acid cycle (TCA; also known as the Krebs cycle or the citric acid cycle) (Nehela and Killiny, 2019, 2022). Additionally, GABA is involved directly in signaling or regulatory mechanisms and indirectly affects plant growth and development throughout their entire lifecycle (from seed to seed) (Ramos-Ruiz et al., 2019). GABA accumulates rapidly in response to abiotic stresses and contributes to responses to biotic stresses through multiple mechanisms. For instance, it increases leaf turgor, and osmolytes, and reduces oxidative damage by stimulation of antioxidants (Sita and Kumar, 2020).

Additionally, β-Aminobutyric acid (BABA) is an isomer of GABA that used to be described as a plant-active xenobiotic molecule, however, it was recently reported as a novel endogenous metabolite that is naturally present in plants (Thevenet et al., 2017; Balmer et al., 2019). It is worth mentioning that endogenous BABA levels were found to be associated with various biotic and abiotic stressors (Baccelli et al., 2017; Thevenet et al., 2017; Balmer et al., 2019), and tightly controlled by the plant’s immune system (Baccelli et al., 2017), and phytohormones (Baccelli and Mauch-Mani, 2016). BABA induces broad-spectrum disease resistance against biotrophic and necrotrophic fungal pathogens (Zimmerli et al., 2000; Ton et al., 2005; Pastor et al., 2013; Buswell et al., 2018; Luna et al., 2020). For instance, BABA application enhanced the resistance of *A. thaliana* against the necrotrophic fungus *Plectosphaerella cucumerina* (Pastor et al., 2013), as well as the biotrophic fungus *Hyaloperonospora arabidopsidis* (Buswell et al., 2018). Moreover, BABA application caused a durable induced resistance in tomato fruit against *Botrytis cinerea, Phytophthora infestans*, and the bacterial pathogen *Pseudomonas syringae* (Luna et al., 2020). Likewise, (*R*)-β-homoserine (RBH; a resistance-inducing analog of GABA/BABA) effectively enhanced the resistance against necrotrophic fungus *P. cucumerina* in *A. thaliana* and against *Botrytis cinerea* in tomato (Buswell et al., 2018). It is worth mentioning that RBH primes different defense pathways against biotrophic and necrotrophic phytopathogens without inhibiting the growth of the host plant and, unlike BABA, RBH does not disturb plant metabolism significantly (Buswell et al., 2018).

Likewise, *ß*-alanine is involved in protecting plants from temperature extremes, hypoxia, drought, heavy metal shock, and some biotic stresses via the induction of lignin biosynthesis and ethylene production in some species (Parthasarathy et al., 2019). Although endogenous GABA concentrations were reported to be higher in legume nodules of common beans (Sita and Kumar, 2020) and *ß*-alanine can accumulate as a generic stress response molecule (Parthasarathy et al., 2019), the physiological, biochemical, and molecular mechanisms behind the potential role(s) of GABA against biotic stress in general, and phytopathogenic fungi specifically, is poorly understood.

We hypothesize that the application of NPAAs such as GABA and *ß*-alanine could be a promising approach for the management to mitigate the impact of white mold disease in common bean cultivation. This study aims to; i) investigate the potential antifungal activity of two NPAAs (GABA and *ß*-alanine) against *S. sclerotiorum*, the causal agent of white mold disease of common beans, *in vitro* and *in vivo* under greenhouse conditions. ii) explore the effects of GABA and *β*-alanine on the growth and development of common bean plants, as well as their influence on disease progression. We believe that the potential role(s) of NPAAs in plant defense and resistance mechanisms are correlated with the activation of enzymatic and non-enzymatic antioxidant defense machinery. Finally, the current study aims to better understand the physio-biochemical mechanisms of NPAAs-induced resistance to develop sustainable and eco-friendly strategies for disease management in common bean cultivation.

## 2 Materials and methods

### 2.1 Plant materials

**Figure 1:**
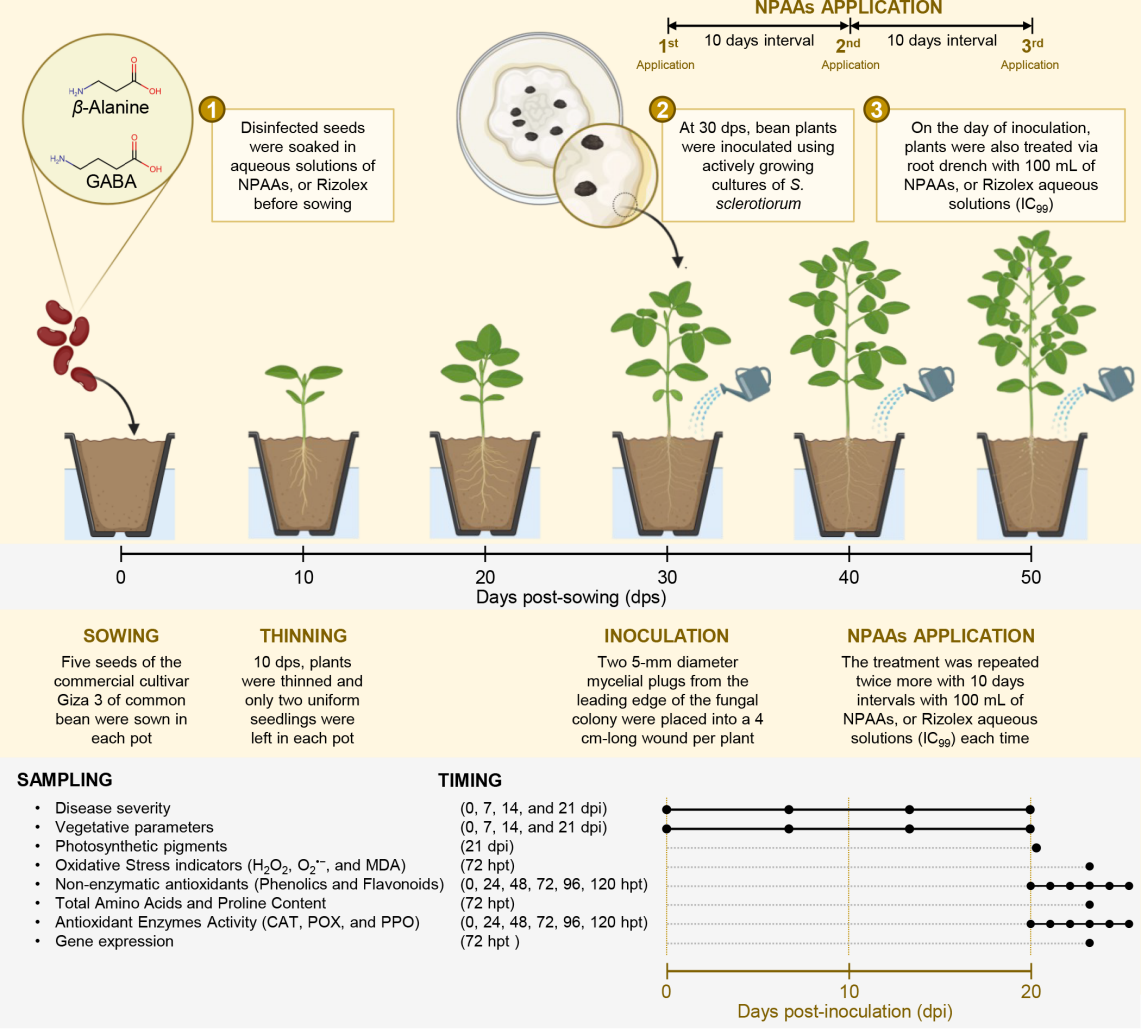
Schematic representation of the experimental design used in this study. Briefly, disinfected seeds were soaked in aqueous solutions (IC_99_ concentration) of NPAAs, or Rizolex before sowing. At 30 days post-sowing (dps), bean plants were inoculated using actively growing cultures of *S. sclerotiorum*. On the day of inoculation, plants were also treated via root drench with 100 mL of NPAAs, or Rizolex aqueous solutions (IC_99_). Moreover, the treatment was repeated twice more at 10-day intervals with 100 mL of NPAAs, or Rizolex aqueous solutions (IC_99_) each time. Sampling points and timing are also presented at the bottom of the schematic representation. dps, Days post-sowing; dpi, Days post-inoculation; and hpt, hours post-last treatment.

The commercial cultivar Giza 3 of common bean (*Phaseolus vulgaris* L. cv. Giza 3) was used as experimental plant material throughout this study. Seeds were kindly provided by the Department of Food Legumes Research, Field Crops Research Institute (FCRI), Agricultural Research Center (ARC), Egypt. The seeds were healthy, uniform, and homologous in size and color. Seeds (five seeds per pot) were sown in plastic pots (30 cm inner diameter and 45 cm in depth) filled with sandy-clay soil (clay and sand at a ratio of 3:1). However, 7-10 days post-sowing and before the first irrigation, plants were thinned and only two uniform seedlings with fully developed trifoliate leaves were left in each pot, then the plants received their first irrigation (**Figure 1**).

### 2.2 Pathogen isolation and identification

Diseased common bean plants showed white mold symptoms (disease severity: 20-30%) and were collected from private fields and commercial farms during the 2020 season. Collected samples showed typical symptoms of white mold including water-soaked lesions with a distinct margin, white cottony fungal mycelial growth on the surfaces of infected aerial tissues, and some of them eventually had a pea-sized black sclerotia on leaves, petiole, stem, and reproductive organs. The collected samples were stored in a plastic box and kept at 4 ± 2°C and 95 ± 2% relative humidity inside the refrigerator till used for the isolation of the causal pathogen.

To isolate the causal agent of white mold disease, infected materials were chopped into small pieces, surface-sterilized with 0.5% NaOCl for three min, then washed several times in sterilized water, and dried between sterilized filter papers to remove the superfluous water. Subsequently, a small piece from the intermediate tissues between healthy and infected ones was directly cultured on potato dextrose agar (PDA) medium and incubated at 25 ± 2°C for 7 days. Furthermore, the hyphal tips method (Brown, 1924) was used to purify the fungal isolate from the mixed or contaminated cultures. Briefly, a small piece of the hyphal tips was removed from a fungal colony using a sterile needle and transferred into a fresh PDA medium and incubated at 25 ± 2°C for 7 days, then identified at the Department of Agricultural Botany, Faculty of Agriculture, Tanta University. Briefly, the purified fungal culture was initially identified based on their cultural morphology characteristics and then the identification was microscopically confirmed as *S. sclerotiorum* based on the morphological features of the mycelium and other observed structures (Barnett and Hunter, 1972). The pathogenicity test was carried out on the commercial cultivar Giza 3 of common bean as described by Viteri et al. (Viteri et al., 2015) to fulfill Koch’s postulates. The isolated fungal strain was virulent and pathogenic on the commercial cultivar Giza 3.

### 2.3 Tested compounds

Two non-proteinogenic amino acids (NPAAs) including γ-amino-butanoic acid (GABA; Chemical formula: C_4_H_9_NO_2_) and 3-aminopropanoic acid (also known as *β*–alanine; Chemical formula: C_3_H_7_NO_2_) purchased from Sigma-Aldrich (Darmstadt, Germany). An appropriate weight of both compounds was initially dissolved in a final volume of 100 mL sterilized water to make a 1000 mg.L^-1^ stock solution that has been diluted and used in all further experiments. Briefly, five concentrations (12.5, 25, 50, 75, and 100 mg.L^-1^) of each compound were tested *in vitro* individually. Moreover, the commercial fungicide Rizolex-T 50% WP (Tolclofos-Methyl 20% + Thiram 30%; KZ–Kafr El Zayat Pesticides and Chemicals, Kafr El-Zayat, Gharbia, Egypt) was used as a positive control throughout the study. On the other hand, sterilized water was used as a negative control (Mock).

### 2.4 *In vitro* antifungal activity of NPAAs against *S. sclerotiorum*

The *in vitro* antifungal activity of both compounds (GABA and *β*–alanine), as well as the commercial fungicide Rizolex was examined using the agar diffusion method (Grover and Moore, 1962). Briefly, an appropriate volume of the stock solution of each compound was mixed with 15 mL PDA medium in a sterilized 9cm-Petri dish to obtain the desired concentration (12.5, 25, 50, 75, and 100 mg.L^-1^) of each compound and five concentrations of Rizolex (2, 4, 6, 8, and 10 mg.L^-1^), while sterilized water was used as a negative control. After media solidification, a 5 mm-diameter mycelial plug of the 5-day-old culture of the pathogenic fungus *S. sclerotiorum* was transferred into the middle of the Petri dish, incubated at 25 ± 2°C until the fungal mycelial growth covered the whole control plate, then the fungal growth was recorded. The experiment was repeated twice with six biological replicates per treatment with the same experimental design as described above. The percentage of radial growth inhibition of *S. sclerotiorum* was calculated using Equation (1):


(1) $$\text{\% Radial growth inhibition = }\frac{\text{C-T}}{\text{C}}\times 100$$

where “C” signifies the diameter of hyphal extension (cm) in the control plates and “T” represents the diameter of mycelial growth in the treatment.

Furthermore, the half-maximal inhibitory concentration (IC_50_) and the inhibitory concentration (IC_99_) were calculated using probit regression analysis (Prentice, 1976) by modeling the probit/logit sigmoid dose–response curves with 95% confidence intervals.

### 2.5 Greenhouse experiments

To evaluate the potential antifungal activity of GABA and *β*–alanine against *S. sclerotiorum* under greenhouse conditions, two pot experiments were carried out in two trials; the first trial was implemented on March 4, 2021, and the second trial on March 3, 2022. Briefly, disinfected seeds of the commercial cultivar Giza 3 of common bean were initially soaked in aqueous solutions of GABA, *β*–alanine, or Rizolex at a final concentration of the IC_99_ (587.57, 541.69, and 26.88 mg.L^−1^, respectively) for 3 hours, then air-dried for one hour prior sowing. In the negative control pots, the seeds were soaked in sterilized water. Subsequently, five seeds were sown in each pot. However, 7-10 days post-sowing and before the first irrigation, plants were thinned and only two uniform seedlings as described above, then the plants received their first irrigation (**Figure 1**). All plants were maintained under greenhouse conditions at 25 ± 2°C, with 75 ± 2% relative humidity (RH), and an 8:16 dark/light cycle. Plants were irrigated twice monthly, or earlier if needed, and fertilized using ammonium sulfate and potassium sulfate according to the recommendations for this cultivar.

One month after sowing (~30 dps), bean plants were inoculated using actively growing cultures of *S. sclerotiorum* that were produced as described above. Briefly, stems of bean plants at the fifth internode were initially cut with a scalpel leaving a 4 cm-long internode intact. Subsequently, two 5-mm diameter mycelial plugs from the leading edge of the fungal colony were placed into each wound per plant (Viteri et al., 2015) (**Figure 1**). Inoculated plants were maintained under the same conditions described above but RH was held >90% by keeping the greenhouse floor wet for at least 7 days post-inoculation (dpi) to encourage infection and disease development.

For NPAAs application, bean plants were watered on the day of inoculation (~30 dps) via root drench with 100 mL of GABA, *β*–alanine, or Rizolex aqueous solutions at a final concentration of the IC_99_ as mentioned above, then NPAAs application was repeated twice more with 10 days intervals (**Figure 1**). Non-treated control was watered with 100 mL of sterilized water. In all cases, 100 mL of the aqueous solution was not enough to fulfill the soil field capacity (SFC) and was completed with sterilized water to reach the SFC. However, the solution that exceeded the SFC, and gravitationally drained, was collected and reapplied to the pots. All common bean plants were maintained under the greenhouse conditions described above. For sampling, three trifoliate leaves were sampled from each biological replicate, from different positions and ages. Collected leaves were homogenized, mixed together (for each biological replicate), and instantly stored at −80°C till further analysis.

### 2.6 Assessment of white mold disease and vegetative parameters

Disease severity was evaluated at 0, 7, 14, and 21 days post-inoculation (dpi; **Figure 1**) (Viteri et al., 2015) using a 1-9 scale of the white mold according to Petzoldt and Dickson scale (Petzoldt and Dickson, 1996) as modified by Terán et al (Terán et al., 2006), then converted into percentages using Equation (2):


(2) $$\text{Disease severity (\%) = }\frac{\text{Total points score}}{\text{Total number of plants }\times \text{ highest score}}\times 100$$

Moreover, the area under the disease progress curve was calculated (Jeger and Viljanen-Rollinson, 2001). Additionally, vegetative growth features of bean plants including plant height (cm), number of branches, and number of leaves were recorded weekly starting at 0, 7, 14, and 21 dpi. Although non-infected control was not included in this study, we just compared the NPAAs-treated plants with non-treated ones which all were infected with *S sclerotiorum*.

### 2.7 Photosynthetic pigment contents

Leaf samples were collected as described above at 21 dpi (~51 dps). Briefly, photosynthetic pigments (chlorophyll *a*, chlorophyll *b*, and carotenoids) were extracted from about 500 mg of homogenized fresh tissues using 80% acetone in the dark for 24 hours at 4°C, then centrifuged and the supernatant was collected. The photosynthetic pigment content was colorimetrically determined in the collected supernatants by measuring the absorbance at different wavelengths (A_470_, A_646_, and A_663_ nm) using a UV-160A spectrophotometer (Shimadzu, Japan) as described by Lichtenthaler, 1987 (Lichtenthaler, 1987). The content (mg g^−1^ FW) of chlorophyll *a*, chlorophyll *b*, and carotenoids were calculated using Equations (3–5) as described by Lichtenthaler, 1987 (Lichtenthaler, 1987).


(3) $$\text{Chlorophyll }a({\text{mg g}}^{-1}\text{FW})\;=\;12.25{\text{A}}_{663}-\;2.79{\text{A}}_{646}$$

(4) $$\text{Chlorophyll }b({\text{mg g}}^{-1}\text{FW})\;=\;21.50{\text{A}}_{646}-\;5.10{\text{A}}_{663}$$

(5) $$\text{Carotenoids }\left({\text{mg g}}^{-1}\text{FW}\right)=\;\frac{1000{\text{A}}_{470}-1.82\text{Chl }a-85.02\text{Chl }b}{198}$$

### 2.8 Oxidative stress indicators

#### 2.8.1 *In situ* histochemical localization hydrogen peroxide

Bean leaves were collected at 72 hours post-last treatment (hpt) for *in situ* histochemical localization of H_2_O_2_ as described by Romero-Puertas et al., 2004 (Romero-Puertas et al., 2004) with slight modifications, as published previously (Nehela et al., 2021). Briefly, 10 fresh leaf discs (~1 cm^2^) were vacuum infiltrated with 0.1% 3,3′-Diaminobenzidine (DAB; Sigma-Aldrich, Darmstadt, Germany) in 10 mM tris buffer (pH 7.8), incubated for one hour under light till the development of brown color, then bleached using a mixture of chloroform and ethanol (1:4; v/v) containing 0.15% trichloroacetic acid (w/v) (Hückelhoven et al., 1999). The intensity of the developed brown color was assessed using the ImageJ image processing package (Fiji version; http://fiji.sc; accessed on 15 June 2023).

#### 2.8.2 *In situ* histochemical localization of superoxide anion

Histochemical localization of superoxide anion (O_2_^•−^) was assessed as described by Adám 1989 (Adám et al., 1989) with slight modifications (Nehela et al., 2021; Nehela and Killiny, 2023a). Briefly, 10 fresh leaf discs (~1 cm^2^) were immersed and vacuum infiltrated with 0.1% nitro blue tetrazolium (NBT; Sigma–Aldrich, Darmstadt, Germany) until the development of a blue/purple color, then bleached, and intensity of developed blue/purple color was assessed the as described above.

#### 2.8.3 Assessment of malondialdehyde

Malondialdehyde (MDA; a product of polyunsaturated fatty acids peroxidation) was determined as described by Du and Bramlage (Du and Bramlage, 1992) with slight modification. Briefly, MDA was extracted from approximately 500 mg of homogenized fresh tissue using 20% trichloroacetic acid (TCA) containing 0.01% butyl hydroxyl toluene (BHT), incubated at 95°C, then centrifuged at 10,000 ×*g* for 10 min. MDA content was colorimetrically determined in the collected supernatants by measuring the absorbance at 532 and 600 (A_532_ and A_600_ nm) using UV-160A spectrophotometer (Shimadzu, Japan), then expressed as nmol g^−1^ FW.

### 2.9 Non-enzymatic antioxidants

Bean leaves were collected at 0, 24, 48, 72, 96, and 120 hours post-last treatment (hpt) for the assessment of non-enzymatic antioxidants as indicated by total soluble phenolics (TSP) and total soluble flavonoids (TSF) (**Figure 1**).

#### 2.9.1 Total soluble phenolic compounds

Total soluble phenolics (TSP) were assessed using the Folin-Ciocalteu reagent as previously described by Kähkönen et al. (Kähkönen et al., 1999) with minor modifications as in our previous study (El-Nagar et al., 2022, 2023). Briefly, about 100 mg of homogenized fresh tissues were extracted with 20 mL of 80% methanol for 24 h in the dark, centrifuged and the supernatants were collected. Subsequently, 200 μl of the supernatant was mixed with one ml of Folin-Ciocalteu reagent (10%), vortexed for 30 seconds, and set on the bench for three min, then 800 μl of 7.5% sodium carbonate was added to the mixture. Subsequently, the mixture was incubated at room temperature for 30 min, and the absorption was measured at 765 nm (A_765_) using a UV-160A spectrophotometer (Shimadzu, Japan). The TSP content is expressed as mg of gallic acid equivalents per gram of fresh weight (mg GAE g^−1^ FW).

#### 2.9.2 Total soluble flavonoids

Total soluble flavonoids (TSF) were examined using Djeridane’s method (Djeridane et al., 2006) with slight modifications as in our previous study (El-Nagar et al., 2022, 2023). Briefly, one ml of methanolic extract (as mentioned above) was mixed with one ml of aluminum chloride (2% in methanol), vigorously mixed, then incubated for 15 min at room temperature. After incubation, the absorption was measured at 430 nm (A_430_) using a UV-160A spectrophotometer (Shimadzu, Japan), and the TSF content was expressed as mg of rutin equivalents per gram of fresh weight (mg RE g-1FW).

#### 2.9.3 Free radical scavenging assay

The free radical scavenging activity of GABA, *β*–alanine, and Rizolex fungicide was spectrophotometrically assessed using 2,2-diphenyl-1-picrylhydrazyl (DPPH) (Reddy et al., 2007) with slight modification as described in our previous studies (El-Nagar et al., 2022). Briefly, 10 µL of aqueous leaf extract was diluted to 100 µL using methanol, mixed with 900 µL of 0.1-mM DPPH methanolic solution, then incubated at room temperature in the dark for 20 min. After incubation, the absorption was measured at 517 nm (A_517_) using a UV-160A spectrophotometer (Shimadzu, Japan). Methanol was used as a blank. The free radical scavenging activity (%) was calculated using Equation (6):


(6) $$\text{Scavenging activity }\left(\%\right)=\;\frac{{\text{A}}_{0}-{\text{A}}_{1}}{{\text{A}}_{0}}\times 100$$

where A_0_ is the absorbance of the control (blank) and A_1_ is the absorbance of the plant extract. Furthermore, the antioxidant efficiency (%) of different plant extracts at 72 hpt with GABA, *β*–alanine, or Rizolex fungicide was calculated using Equation (7):


(7) $$\text{Antioxidant efficiency }\left(\%\right)=\;\frac{\text{Scavenging activity }\left(\%\right)\text{ of treatment}}{\text{Scavenging activity }\left(\%\right)\text{ of control}}\times 100$$

### 2.10 Total amino acids and proline content

Bean leaves were collected at 72 hpt for the assessment of total amino acids and proline content. The total free amino acids (TAA) were quantified using a modified ninhydrin reagent (Yokoyama and Hiramatsu, 2003) with efficient improvements by Sun et al (Sun et al., 2006). Briefly, TAA was extracted from 100 mg ground frozen tissues using a buffer composed of acetic acid and sodium acetate (pH 5.4), then centrifuged at 1000× g for ten min. Subsequently, 200 μL of the supernatant was reacted with 200 μL of ninhydrin (2%) and 200 μL of pyridine (10%), vigorously mixed, then incubated for 30 min in a boiling-water water bath. After incubation, the mixture was diluted using distilled water as needed, then the absorption was measured at 580 nm (A_580_) using a UV-160A spectrophotometer (Shimadzu, Japan).

Moreover, proline content was determined using Bates’s method (Bates et al., 1973). Briefly, proline was extracted using 5 mL of 3% sulfosalicylic acid, then centrifuged and the supernatant was collected. Subsequently, about 1 mL of the supernatant was mixed with 2 mL of a dual of glacial acetic acid and ninhydrin reagent, then incubated at 90°C for 45 min. After incubation, the mixture was cooled and 5 mL toluene was added, then the absorption of the upper phase was measured at 520 nm (A_520_) using a UV-160A spectrophotometer (Shimadzu, Japan). Analytical grade proline was used to prepare the standard curve and total free amino acids and proline content were expressed as mg g^−1^ FW.

### 2.11 Antioxidant enzymes activity

To determine the enzymatic activity of antioxidant-associated enzymes, Bean leaves were collected at 0, 24, 48, 72, 96, and 120 hpt (**Figure 1**). Subsequently, about 500 mg of homogenized fresh tissues were extracted using 3 mL of 50 mM Tris buffer (pH 7.8) containing 1 mM EDTA-Na_2_ and 7.5% polyvinylpyrrolidone (PVP), centrifuged at 10000× *g* for 20 min under cooling (4°C), and the supernatant (crude enzyme extract) was collected (El-Nagar et al., 2023). Subsequently, the supernatant was divided into aliquots as follows.

#### 2.11.1 Catalase activity

The enzymatic activity of catalase (CAT) was assessed according to the method of Aebi (Aebi, 1984) with slight modifications (El-Nagar et al., 2023). Briefly, an aliquot of 50 µL of crude enzyme extract was mixed with a reaction mixture containing 2 mL of 0.1 M sodium phosphate buffer (pH 6.5) and 100 μL of 269 mM H_2_O_2_ solution. CAT activity was colorimetrically measured by following the decomposition of H_2_O_2_ at 240 nm (A_240_) using a UV-160 spectrophotometer (Shimadzu, Japan).

#### 2.11.2 Guaiacol-dependent peroxidases activity

The enzymatic activity of guaiacol-dependent peroxidases (POX) was determined using the method of Harrach et al. (Harrach et al., 2008) with slight modifications (El-Nagar et al., 2023). Briefly, an aliquot of 10 µL of crude enzyme extract was mixed with a reaction mixture containing 2.2 mL of 100 mM sodium phosphate buffer (pH 6.0), 100 µL guaiacol, and 100 µL of 12 mM H_2_O_2_. POX activity was colorimetrically measured by following the increase in the absorption at 436 nm (A_436_) using a UV-160 spectrophotometer (Shimadzu, Japan).

#### 2.11.3 Polyphenol oxidase activity

The enzymatic activity of Polyphenol oxidase (PPO) was determined according to the method of Malik and Singh (Malik and Singh, 1980) with slight modifications (El-Nagar et al., 2023). Briefly, an aliquot of 100 µL of crude enzyme extract was mixed with 3 mL catechol solution (0.01 M), freshly prepared in 0.1 M phosphate buffer (pH 6.0). PPO activity was colorimetrically measured by recording the fluctuations in the absorbance at 495 nm (A_495_) every 30 seconds for 3 min.

### 2.12 Gene expression of antioxidant-associated genes

The transcript levels of three antioxidant-associated genes, including peroxisomal catalase (*PvCAT1*; GenBank accession number: KF033307.1), copper/zinc superoxide dismutase (*PvCuZnSOD1*; GenBank accession number: KF569535.1), and glutathione reductase (*PvGR*; GenBank accession number: KY195009.1) were assessed from common bean leaves at 72 hpt after last treatment using real-time RT-PCR. Briefly, RNA was extracted using a Simply P Total RNA Extraction Kit (catalog number BSC52S1), according to the manufacturer’s procedure. Then, cDNA was synthesized using a TOP script™ cDNA Synthesis Kit as described in the manufacturer’s protocol. The primer sequences for the three genes mentioned above are previously described by ElSayed et al (ElSayed et al., 2021). Actin was used as a housekeeping gene (El-Nagar et al., 2022), and the 2^−ΔΔCT^ method was used for the calculation of relative gene expression (Livak and Schmittgen, 2001).

### 2.13 Statistical analyses

All experiments were arranged in a completely randomized design (CRD) with six biological replicates per treatment and five pots (two plants per pot) for each replicate. Data were statistically analyzed using the analysis of variance (ANOVA), followed by the Tukey-Kramer honestly significant difference (HSD) test (*p* ≤ 0.05). For the *in vitro* experiments, probit analysis was used to calculate the IC_50_ and IC_99_ with 95% confidence intervals (Prentice, 1976).

## 3 Results

### 3.1 *In vitro* antifungal activity of NPAAs against *S. sclerotiorum*

**Figure 2:**
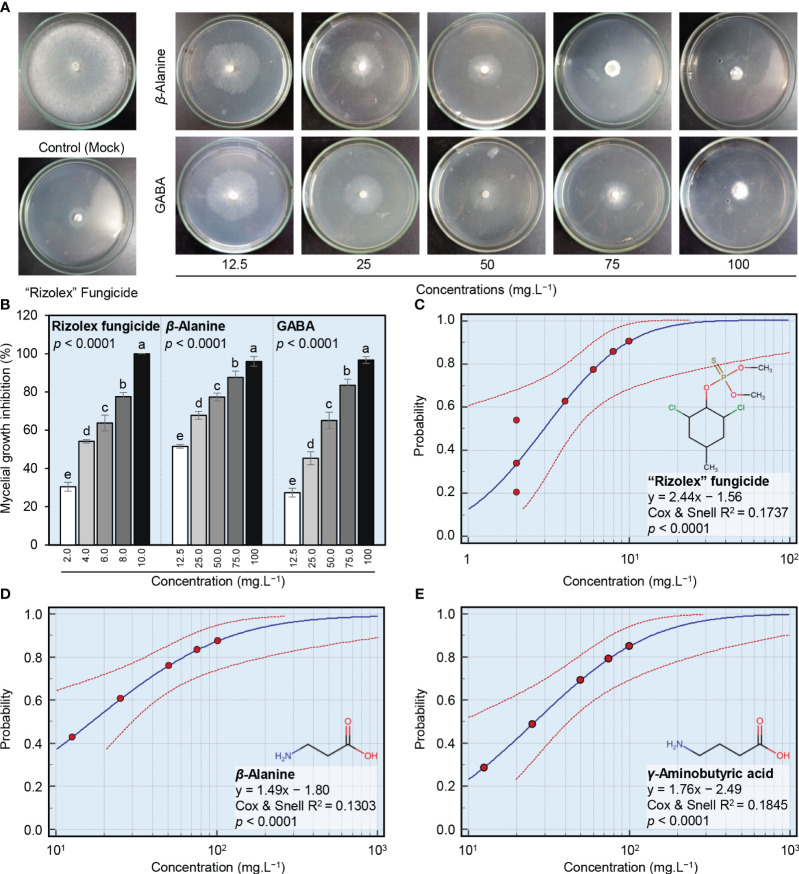
*In vitro* antifungal activity of two NPAAs (GABA and *ß*-alanine) against *S. sclerotiorum*, the causal agent of white mold disease of common bean. **(A)** Antifungal activity of GABA and *ß*-alanine against *S. sclerotiorum*, **(B)** Mycelial growth inhibition (%) of *S. sclerotiorum* after the treatment with different concentrations of GABA and *ß*-alanine (12.5, 25, 50, 75, and 100 mg.L^−1^). Bars represent the average of six biological replicates (*n* = 6), whereas whiskers represent the standard deviation (means ± SD). Different letters indicate statistically significant differences between treatments (*p<* 0.05). **(C–E)** Probit regression analysis of the inhibition effects of the commercial fungicide “Rizolex”, *ß*-alanine, and GABA, respectively, against *S. sclerotiorum*. The probit regression lines are presented as blue solid lines, whereas the confidence intervals (95%) are edged with dotted red lines.

The antifungal activity of different concentrations of GABA, *ß*-alanine, and Rizolex fungicide against *S. sclerotiorum*, the causal agent of white mold disease of common bean was examined *in vitro* (**Figure 2**). Generally, both NPAAs (GABA and *ß*-alanine) showed a strong dose-dependent fungistatic activity and progressively inhibited the mycelial radial growth of *S. sclerotiorum* (**Figure 2A**). Although the highest concentration of “Rizolex” fungicide (10 mg.L^-1^) completely inhibited the mycelial growth of *S. sclerotiorum* (100% mycelial growth inhibition), the highest concentrations of *ß*-alanine and GABA (100 mg.L^-1^) were almost similar to it and significantly inhibited the mycelial growth of *S. sclerotiorum* by 96.0 and 97.7%, respectively (**Figure 2B**). Moreover, probit analysis showed that the regression lines of “Rizolex” fungicide (**Figure 2C**), as well as *ß*-alanine (**Figure 2D**) and GABA (**Figure 2E**), displayed the same positive upward trend. Regardless of the “Rizolex” fungicide, *ß*-alanine had a lower slope value (y = 1.49x − 1.80, Cox and Snell R^2^ = 0.1303, and *p*< 0.0001; **Figure 2D**) than GABA (y = 1.76x − 2.49, Cox and Snell R^2^ = 0.1845, and *p*< 0.0001; **Figure 2E**). Accordingly, *ß*-alanine had a lower IC_50_ (16.11 mg.L^-1^) than GABA (25.88 mg.L^-1^) (**Table 1**). Collectively, these findings suggest that both NPAAs (GABA and *ß*-alanine) have potent antifungal activity against *S. sclerotiorum*, the causal agent of white mold disease of common bean.

**Table 1:** The half-maximal inhibitory concentration (IC_50_) and IC_99_ values (mg.L^-1^) of two NPAAs (GABA and *ß*-alanine) and the commercial fungicide “Rizolex” against *Sclerotinia sclerotiorum*.

Compound	IC_50_ (mg.L^−1^)	95% Confidence Interval		IC_99_ (mg.L^−1^)	95% Confidence Interval		Overall Model Fit		
		Lower	Upper		Lower	Upper	χ^2^	p-Value	Cox & Snell R^2^
*β*-Alanine	16.11	2.09	28.95	587.57	188.18	113337.64	502.67	< 0.0001	0.1303
*γ*-Aminobutyric acid	25.88	8.61	41.95	541.69	194.79	27942.81	734.18	< 0.0001	0.1845
Rizolex “Fungicide”	2.98	0.30	4.73	26.88	11.40	67318.79	572.22	< 0.0001	0.1737

### 3.2 NPAAs reduced the development of white mold disease on common beans

**Figure 3:**
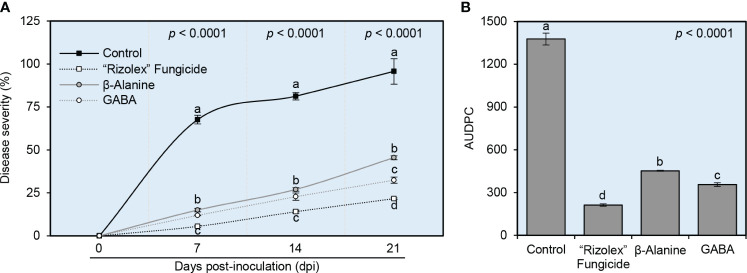
Effect of the exogenous application of two NPAAs (GABA and *ß*-alanine) on the evaluation of white mold disease of common beans caused by *S. sclerotiorum* under greenhouse conditions. **(A)** Disease progress curves of white mold disease of common beans at 0, 7, 14, and 21 days post-inoculation (dpi) with *S. sclerotiorum*. **(B)** The area under the disease progress curve (AUDPC) of white mold disease of common beans after the treatment with GABA or *ß*-alanine. Values represent the means ± standard deviation (means ± SD) of six biological replicates (*n* = 6). Different letters indicate statistically significant differences between treatments (*p*< 0.05).

Generally, both NPAAs (GABA and *ß*-alanine) reduced the temporal progression of white mold disease severity in common beans under greenhouse conditions (**Figure 3**). Briefly, although the white mold disease severity was increased progressively in mock-treated plants (negative control), both GABA and *ß*-alanine significantly decreased the disease severity (%) at 7 dpi and till the end of the experiment at 21 dpi (**Figure 3A**). It is worth mentioning that, although there were no significant differences in disease severity (%) between both NPAAs at 7 and 14 dpi, GABA was more effective and had lower disease severity (32.38 ± 1.80%) than *ß*-alanine (45.47 ± 1.09%) at 21 dpi (**Figure 3A**). Likewise, GABA-treated plants had a lower AUDPC value (356.45 ± 12.42) than *ß*-alanine (453.15 ± 3.34) which were significantly lower than the mock-treated control (1377.18 ± 41.94) (**Figure 3B**). Collectively, these findings suggest that exogenous application of both NPAAs (GABA and *ß*-alanine) weakens the development of white mold disease in common bean plants.

### 3.3 NPAAs enhance the vegetative growth of *S. sclerotiorum*-infected bean plants

**Figure 4:**
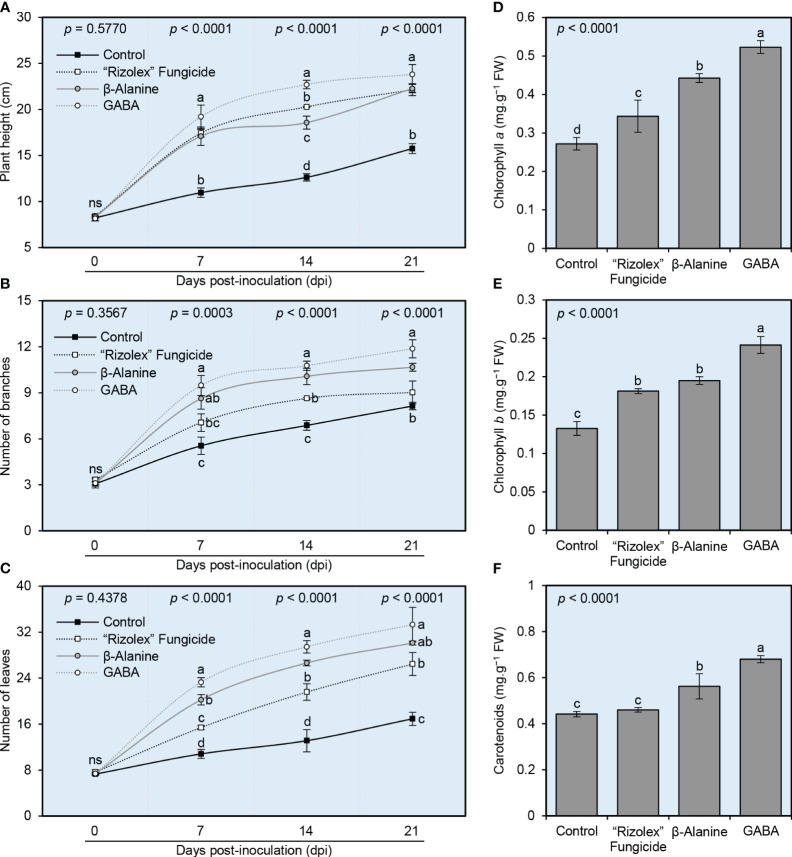
Effect of the exogenous application of two NPAAs (GABA and *ß*-alanine) on the growth variables and photosynthetic pigments in the leaves of *S. sclerotiorum*-infected common beans under greenhouse conditions. **(A)** Plant height (cm), **(B)** number of branches per plant, and **(C)** number of leaves per plant at 0, 7, 14, and 21 days post-inoculation (dpi) with *S. sclerotiorum*. **(D)** Chlorophyll *a* content (mg.g^−1^ FW), **(E)** chlorophyll *b* content (mg.g^−1^ FW), and **(F)** total carotenoids content (mg g^−1^ FW) at 21 dpi (~50 dps). Values represent the means ± standard deviation (means ± SD) of six biological replicates (*n* = 6). Different letters indicate statistically significant differences between treatments (p< 0.05).

Although non-infected control was not included in this study, we just compared the NPAAs-treated plants with non-treated ones which all were infected with *S sclerotiorum*. It is worth mentioning that no significant differences were observed between different treatments in terms of plant height (**Figure 4A**), number of branches (**Figure 4B**), and number of leaves (**Figure 4C**) at 0 dpi (~30 dps). However, the exogenous application of both NPAAs (GABA and *ß*-alanine), as well as the commercial fungicide “Rizolex” significantly increased the vegetative growth of *S. sclerotiorum*-infected bean plants at 7 dpi till the end of the experiment (~21 dpi) with superiority for GABA application followed by *ß*-alanine. Briefly, GABA-treated plants had the highest plant height (23.81 ± 1.07 cm), number of branches (11.87 ± 0.59), and number of leaves (33.29 ± 3.01), followed by *ß*-alanine-treated plants (22.27 ± 0.45 cm, 10.67 ± 0.25, and 30.11 ± 0.33; respectively) compared with the mock-treated control plants (15.76 ± 0.53 cm, 8.14 ± 0.24, and 16.91 ± 1.15; respectively). In other words, NPAAs produced the greatest improvements in plant height, with an average increase of 41-51% over the control group (**Figure 4A**). Likewise, both NPAAs improved the number of branches (with average increases of 31-46%; **Figure 4B**) and the number of leaves (with average increases of 78-97%; **Figure 4C**) over the control group. Collectively, these findings suggest that the exogenous application of both NPAAs (GABA and *ß*-alanine) has no potential phytotoxicity on the treated bean plants.

### 3.4 NPAAs boost the content of photosynthetic pigments of infected plant

Generally, the exogenous application of both NPAAs significantly increased the content of photosynthetic pigments in the leaves of treated *S. sclerotiorum*-infected bean plants including chlorophyll *a* (**Figure 4D**), chlorophyll *b* (**Figure 4E**), and total carotenoids (**Figure 4F**). It is worth noting that GABA application had the highest levels of all photosynthetic pigments including chlorophyll *a* (0.52 ± 0.02 mg.g^−1^ FW), chlorophyll *b* (0.24 ± 0.01 mg.g^−1^ FW), and total carotenoids (0.68 ± 0.02 mg.g^−1^ FW), followed by *ß*-alanine-treated plants (0.44 ± 0.01, 0.20 ± 0.01, and 0.56 ± 0.05 mg.g^−1^ FW; respectively) which both were significantly higher than the mock-treated control (0.27 ± 0.02, 0.13 ± 0.01, and 0.44 ± 0.01 mg.g^−1^ FW; respectively).

### 3.5 NPAAs alleviate the oxidative stress of *S. sclerotiorum*-infected bean plants

**Figure 5:**
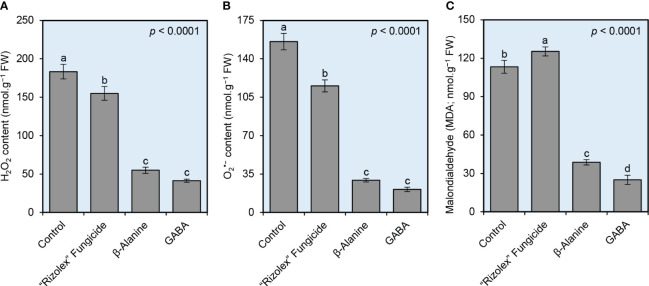
Effect of the exogenous application of two NPAAs (GABA and *ß*-alanine) on the oxidative stress indicators in the leaves of *S. sclerotiorum*-infected common beans at 72 hpt under greenhouse conditions. **(A)** hydrogen peroxide (H_2_O_2_; nmol g^−1^ FW), **(B)** superoxide anion (O_2_^•−^; nmol g^−1^ FW), and **(C)** Malondialdehyde (MDA; nmol g^−1^ FW) at 72 hours post-last treatment (hpt). Bars represent the average of six biological replicates (*n* = 6), whereas whiskers represent the standard deviation (means ± SD). Different letters indicate statistically significant differences between treatments (*p<* 0.05).

Although the infection with *S. sclerotiorum* markedly increased the oxidative stress in infected bean plants as expressed by the levels of hydrogen peroxide (H_2_O_2_; 183.33 ± 9.61 nmol.g^−1^ FW, **Figure 5A**), superoxide anion (O_2_^•−^; 155.67 ± 7.51 nmol.g^−1^ FW, **Figure 5B**), and malondialdehyde (MDA; 113.33 ± 5.03 nmol.g^−1^ FW, **Figure 5C**), both NPAAs (GABA and *ß*-alanine) significantly reduced the levels of the three studied oxidative Stress indicators. Briefly, GABA-treated plants had the lowest H_2_O_2_ (41.33 ± 2.08 nmol.g^−1^ FW) and O_2_^•−^ (21.00 ± 2.00 nmol.g^−1^ FW) levels as shown by DAB-based and NBT-based *in situ* histochemical localization, respectively, followed by *ß*-alanine-treated plants (55.00 ± 4.00 and 29.33 ± 1.53 nmol.g^−1^ FW; respectively). Moreover, although the application of the commercial fungicide “Rizolex” significantly increased the lipid peroxidation in treated infected plants as indicated by higher levels of MDA (125.33 ± 3.51 nmol.g^−1^ FW) compared with mock-treated plants (113.33 ± 5.03 nmol.g^−1^ FW), exogenous application of GABA or *ß*-alanine meaningly reduced the accumulation of MDA (25.00 ± 3.61 and 38.67 ± 2.08 nmol.g^−1^ FW, respectively) in treated infected plants (**Figure 5C**). Taken together, these findings suggest that exogenous treatment with GABA or *ß*-alanine alleviates the oxidative stress in *S. sclerotiorum*-infected bean leaves.

### 3.6 NPAAs enhanced the profile of non-enzymatic antioxidants of *S. sclerotiorum*-infected bean plants

**Figure 6:**
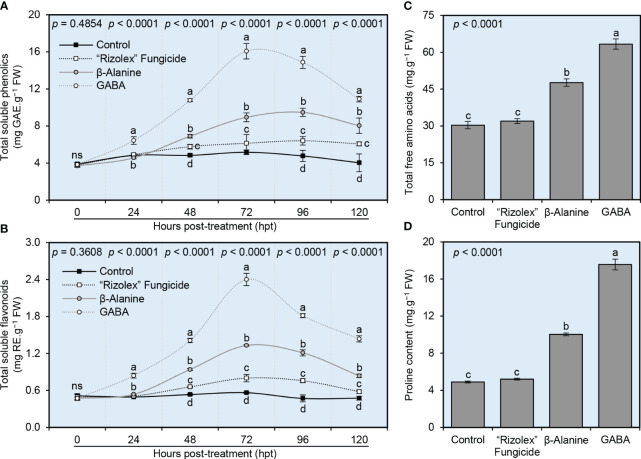
Effect of the exogenous application of two NPAAs (GABA and *ß*-alanine) on the biochemical profile of *S. sclerotiorum*-infected common beans under greenhouse conditions. **(A)** total soluble phenolics (mg GAE g^−1^ FW), **(B)** total soluble flavonoids (mg RE g^−1^ FW) at 0, 24, 48, 72, 96, 120 hours post-last treatment (hpt), **(C)** Total free amino acids (mg.g^−1^ FW) at 72 hpt, and **(D)** Proline content (mg.g^−1^ FW) at 72 hpt. Values represent the means ± standard deviation (means ± SD) of six biological replicates (*n* = 6). Different letters indicate statistically significant differences between treatments (*p*< 0.05).

To better understand the biochemical mechanisms behind how NPAAs (GABA and *ß*-alanine) alleviate the oxidative stress in *S. sclerotiorum*-infected plants, the endogenous levels of total soluble phenolics and total soluble flavonoids (as indicators of non-enzymatic Antioxidants) were investigated. Generally, both compounds enhanced the profile of total soluble phenolics (**Figure 6A**) and total soluble flavonoids (**Figure 6B**) of infected plants and reached their highest peak at 72 hours post-treatment (hpt). Briefly, although the profile of both total soluble phenolics and total soluble flavonoids did not change significantly in mock-treated plants over 120 hpt, exogenous application of GABA progressively increased the levels of total soluble phenolics and total soluble flavonoids to reach their highest peak at 72 hpt (16.07 ± 0.83 mg GAE.g^−1^ FW and 2.40 ± 0.10 mg RE.g^−1^ FW, respectively), followed by *ß*-alanine treated plants at the same time point (8.93 ± 0.47 mg GAE.g^−1^ FW and 1.33 ± 0.01 mg RE.g^−1^ FW, respectively) then declined again at 96 and 120 hpt (**Figures 6A, B**).

### 3.7 NPAAs boosted the profile of total amino acids and proline content of *S. sclerotiorum*-infected bean plants

The application of the commercial fungicide “Rizolex” affected neither the total amino acids (32.00 ± 1.00 mg.g^−1^ FW; **Figure 6C**) nor proline content (5.20 ± 0.10 mg.g^−1^ FW; **Figure 6D**) compared with the mock-treated control (30.33 ± 1.53 and 4.90 ± 0.10 mg.g^−1^ FW, respectively). However, exogenous application of GABA or *ß*-alanine significantly increased the accumulation of total amino acids (63.33 ± 2.08 and 47.67 ± 1.53 mg.g^−1^ FW, respectively; **Figure 6C**) and proline content (17.57 ± 0.59 and 10.03 ± 0.15 mg.g^−1^ FW, respectively; **Figure 6D**) with superiority for GABA application followed by *ß*-alanine.

### 3.8 NPAAs improved the scavenging activity and antioxidant efficiency of *S. sclerotiorum*-infected bean plants

**Figure 7:**
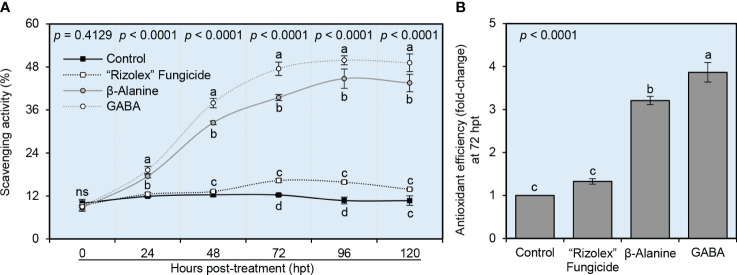
Effect of the exogenous application of two NPAAs (GABA and *ß*-alanine) on the scavenging activity and antioxidant efficiency of *S. sclerotiorum*-infected common beans under greenhouse conditions. **(A)** Scavenging activity (%) at 0, 24, 48, 72, 96, 120 hours post-last treatment (hpt), and **(B)** Antioxidant efficiency (fold-change) at 72 hpt. Values represent the means ± standard deviation (means ± SD) of six biological replicates (*n* = 6). Different letters indicate statistically significant differences between treatments (*p*< 0.05).

The potential of both NPAAs (GABA and *ß*-alanine) to improve the scavenging activity and antioxidant efficiency of *S. sclerotiorum*-infected bean plants was investigated *in vitro* (**Figure 7**). Exogenous application of both GABA and *ß*-alanine progressively enhanced the scavenging activity of leaf extract from *S. sclerotiorum*-infected plants over a time course of 120 hpt (**Figure 7A**). In contrast, the positive control “Rizolex” fungicide showed slight scavenging activity only at 72 and 96 hpt. Accordingly, GABA-treated plants had the highest antioxidant efficiency (more than 3.8-fold change), followed by *ß*-alanine-treated ones (approximately 3.2-fold change) when calculated at 72 hpt compared with the mock-treated control (**Figure 7B**).

### 3.9 NPAAs enhanced the profile of enzymatic antioxidants of *S. sclerotiorum*-infected bean plants

**Figure 8:**
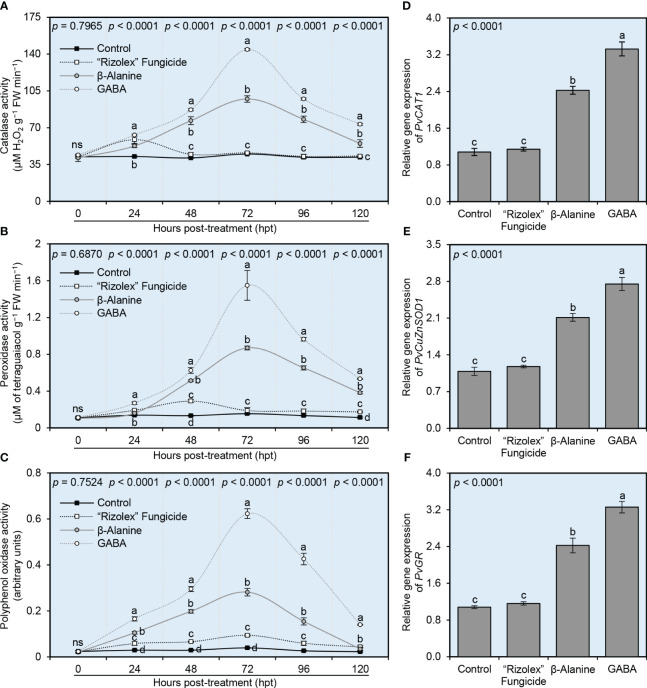
Effect of the exogenous application of two NPAAs (GABA and *ß*-alanine) on the antioxidant defense-related enzymes of *S. sclerotiorum*-infected common beans under greenhouse conditions. **(A)** catalase activity (μM H_2_O_2_ g^−1^ FW min^−1^), **(B)** peroxidase activity (μM Tetraguaiacol g^−1^ FW min^−1^), and **(C)** polyphenol oxidase activity (Arbitrary units) at 0, 24, 48, 72, 96, 120 hours post-last treatment (hpt), **(D–F)** Relative gene expression of peroxisomal catalase (*PvCAT1*), copper/zinc superoxide dismutase (*PvCuZnSOD1*), and glutathione reductase (*PvGR*), respectively, at 72 hpt. Values represent the means of six biological replicates (*n* = 6), while whiskers reflect the standard deviation (means ± SD). Different letters indicate statistically significant differences between treatments (*p*< 0.05).

To better understand the physiological mechanisms behind how NPAAs (GABA and *ß*-alanine) alleviate the oxidative stress in *S. sclerotiorum*-infected plants, enzymatic activities of three antioxidant-associated enzymes included catalase (CAT; **Figure 8A**), peroxidases (POX; **Figure 8B**) and polyphenol oxidase (PPO; **Figure 8C**) were investigated. Briefly, the enzymatic activities of these three antioxidant-associated enzymes considerably heightened after the application of both NPAAs (GABA and *ß*-alanine), but not the commercial fungicide “Rizolex”. The enzymatic activity of CAT progressively accumulated to reach its highest level at 72 hpt with GABA (144.33 ± 7.57 μM H_2_O_2_ g^−1^ FW min^−1^) or *ß*-alanine (97.33 ± 3.06 μM H_2_O_2_ g^−1^ FW min^−1^) then decreased at 96 and 120 hpt (**Figure 8A**). Likewise, POX activity was also gradually elevated to reach its highest level at 72 hpt with GABA (1.55 ± 0.16 μM of tetraguaiacol g^−1^ FW min^−1^) or *ß*-alanine (0.87 ± 0.02 μM of tetraguaiacol g^−1^ FW min^−1^), however, it rapidly declined at 96 and 120 hpt (**Figure 8B**). Similarly, the enzymatic activity of PPO had the same profile as CAT and POX and reached its highest peak at 72 hpt with GABA (0.62 ± 0.02 arbitrary units) or *ß*-alanine (0.28 ± 0.02 arbitrary units), then swiftly decayed at 96 and 120 hpt (**Figure 8C**).

Furthermore, the transcript levels of three antioxidant-related genes included peroxisomal catalase (*PvCAT1*; **Figure 8D**), copper/zinc superoxide dismutase (*PvCuZnSOD1*; **Figure 8E**), and glutathione reductase (*PvGR*; **Figure 8F**) were examined in common bean leaves at 72 hpt. In general, the commercial fungicide “Rizolex” did not alter the expression levels of these three genes. However, the transcript levels of the three tested genes (*PvCAT1*, *PvCuZnSOD1*, and *PvGR*) were significantly increased after the treatment with GABA (3.33-, 2.75-, and 3.26-fold change, respectively), followed by *ß*-alanine (2.43-, 2.11-, and 2.42- fold change, respectively) compared with mock-treated control.

## 4 Discussion

White mold disease, caused by the necrotrophic fungus *S. sclerotiorum*, poses a noteworthy threat to the common bean industry worldwide (Schwartz et al., 2005). The broad host range of *S. sclerotiorum*, its adaptability to varied environments, and its ability to produce long-lasting sclerotia that can survive in the soil for prolonged periods make it a formidable adversary (Schwartz et al., 2005; Figueirêdo et al., 2010; Elsheshtawi et al., 2017). Although the biological control of *S. sclerotiorum* has been experimentally proofed recently (Ali and Atallah, 2020; Atallah and Yassin, 2020; Atallah et al., 2022), conventional control measures mainly rely on chemical fungicides, which have several concerns about their disadvantageous environmental effect and the potential emergence of fungicide-resistant strains (Zubrod et al., 2019; Yong et al., 2022). Therefore, it is necessary to introduce some eco-friendly alternatives to control *S. sclerotiorum* particularly, and soil-borne phytopathogens in general. NPAAs have emerged as potent fungistatic agents that could be promising eco-friendly alternatives to control fungal phytopathogens (Marcucci et al., 2010; Justyna and Ewa, 2013; de Oliveira et al., 2019; Guo et al., 2022), as well as bacterial ones (Nehela and Killiny, 2019; Nehela and Killiny, 2023a, b).

Our findings showed that both NPAAs (GABA and *ß*-alanine) exhibited a strong dose-dependent fungistatic activity and progressively inhibited the mycelial growth of *S. sclerotiorum*, with superiority for GABA. The potential antifungal activity of NPAAs was reported previously (Deng et al., 2023). For instance, GABA and two of its analogs including *α*-aminobutyric acid (AABA) and *β*-aminobutyric acid (BABA) inhibited spore germination of *P. expansum in vivo* at a concentration of 100 mg.L^−1^ (Fu et al., 2017). Moreover, BABA was reported to protect more than 40 plant species against approximately 80 phytopathogens and herbivory, including a virus, Protista, bacteria, oomycetes, fungi, nematodes, and arthropods (Cohen et al., 2016). For example, it showed direct *in vitro* antifungal activity against *S. sclerotiorum* (Marcucci et al., 2010)*, Leptosphaeria maculans* (Šašek et al., 2012), *Penicillium digitatum* (Elsherbiny et al., 2021), and *Rhizoctonia solani* (Deng et al., 2023). It is worth mentioning that the antifungal activity of BABA is better studied than GABA (Šašek et al., 2012; Cohen et al., 2016; Thevenet et al., 2017). However, GABA was reported to be accumulated as a part of the metabolomic responses of soybean plants against *S. sclerotiorum* (de Oliveira et al., 2019), as well as grapevine response against *Neofusicoccum parvum*. Additionally, GABA plays a positive role in the host response to bacterial phytopathogens such as *Agrobacterium tumefaciens* (Chevrot et al., 2006), *Pseudomonas syringae* (Park et al., 2010; Deng et al., 2020), and ‘*Candidatus* Liberibacter Asiaticus’ (Killiny and Nehela, 2017; Nehela and Killiny, 2019, 2022; Nehela and Killiny, 2023a, b). In other words, augmented GABA shunt activity was reported to be associated with plant defense against *Agrobacterium* in tobacco (Chevrot et al., 2006), *Botrytis cinerea* in tomato (Seifi et al., 2013), and ‘*Candidatus* Liberibacter Asiaticus’ in citrus (Killiny and Nehela, 2017; Nehela and Killiny, 2019, 2022; Nehela and Killiny, 2023a, b).

While the precise biochemical, physiological, and molecular mechanisms underlying the antifungal activity of GABA and *ß*-alanine remain poorly investigated, numerous potential modes of action have been proposed. GABA may interfere with fungal cell wall synthesis and disrupt cellular signaling pathways (Yu et al., 2023). For example, GABA inhibited the mycelial growth of *Alternaria alternata*, the causal agent of black spot disease in tomatoes, via downregulating several genes related to sporulation, toxin synthesis, and cell wall degradation enzymes (Yu et al., 2023). Likewise, BABA significantly inhibited mycelial growth, spore germination, and germ tube elongation of *P. digitatum*, the causal agent of green mold in orange fruit, via increasing the relative electrical conductivity, as well as malondialdehyde (MDA) levels in the mycelium, but caused a considerable reduction in the ergosterol content in the plasma membrane and the total lipid content of the mycelium (Elsherbiny et al., 2021). Moreover, electron microscopy examination showed that both GABA and BABA negatively affect the mycelial structure of treated fungal pathogens as indicated by shrunken, distorted, and collapsed mycelia (Elsherbiny et al., 2021; Yu et al., 2023). This supports the hypothesis that GABA interferes with fungal cell wall synthesis. However, further studies are required to investigate the mechanisms by which GABA and *ß* -alanine antagonists the fungal mycelial growth.

Our greenhouse experiments showed that the application of NPAAs via seed soaking and then via three root drench applications enhanced the resistance of common bean plants to *S. sclerotiorum* infection. Both NPAAs have effectively reduced disease severity, augmented the growth, and enhanced the photosynthetic pigment content of treated common bean plants under the infection with *S. sclerotiorum* and compared with the non-treated infected ones. It is worth noting that neither GABA nor *ß*-alanine produced any phytotoxicity in treated bean plants as shown by enhanced growth performance. Similarly, the GABA analog BABA significantly reduced the disease severity/incidence of white mold in artichokes caused by *S. sclerotiorum* (Marcucci et al., 2010), green mold in orange fruits caused by *P. digitatum* (Elsherbiny et al., 2021), and sheath blight of rice caused by *R. solani* (Deng et al., 2023). However, the biochemical and physiological mechanisms behind these roles are poorly understood. This might be due to the modulation of multiple metabolic pathways and redox status (Nehela and Killiny, 2023a). For instance, the tolerance of citrus plants against the bacterial pathogen ‘*Ca*. Liberibacter asiaticus’ was enhanced by the positive regulation of the GABA shunt and associated pathways such as amino acids (Killiny and Nehela, 2017; Nehela and Killiny, 2023a), polyamines (Nehela and Killiny, 2020; Nehela and Killiny, 2023a), TCA cycle, phytohormones (Nehela and Killiny, 2023b), and other stress-associated metabolites (Nehela and Killiny, 2020; Nehela and Killiny, 2023a). The dual function of GABA as a metabolite and as a signaling component enables plants to cope with different conditions. Exogenously applied GABA triggers similar effects to the endogenous molecule and may offer the potential to improve the overall vigor of plants (Ramos-Ruiz et al., 2019). For example, GABA was increased in the *S. sclerotiorum*-infected stems, but not in the leaves of soybean plants and it might be involved in the defense response of soybean plants against *S. sclerotiorum* infection (de Oliveira et al., 2019). GABA can act as a source of nitrogen and carbon to produce defensive specialized metabolites, such as phytoalexins and phenolic compounds in soybeans. Furthermore, GABA primarily processes reactive oxygen species (ROS) that are generated during the oxidative burst and the hypersensitive response (de Oliveira et al., 2019) indirectly through its role in plant stress responses. While GABA itself does not directly scavenge ROS molecules, it acts as a signaling molecule that triggers downstream pathways involved in ROS processing and antioxidant defense mechanisms.

Another hypothesis that could explain how NPAAs effectively reduced disease severity is due to their ability to modulate the redox status within infected plants. Our findings showed that *S. sclerotiorum* infection triggers oxidative stress in bean plants, leading to the overproduction of reactive oxygen species (ROS) such as H_2_O_2_ and O_2_^•−^, as well as lipid peroxidation (MDA). These ROS are extremely toxic, inflict cellular damage, negatively affect proteins, lipids, carbohydrates, and DNA which ultimately results in oxidative stress (Gill and Tuteja, 2010), and contribute to disease progression (Bandyopadhyay et al., 1999; Torres et al., 2006). However, it is worth mentioning that the application of both NPAAs effectively reduced ROS levels and alleviated oxidative stress in *S. sclerotiorum*-infected bean plants. These beneficial effects are likely attributed to the ability of GABA and *ß*-alanine to alleviate oxidative stress and bolster a multilayered antioxidant defense system to neutralize the damaging consequence of ROS and to preserve their homeostasis within *S. sclerotiorum*-infected plants. This multilayered antioxidant defense incorporates two main mechanisms, enzymatic and non-enzymatic antioxidant defense machinery (Ahmad et al., 2010; Gill and Tuteja, 2010; Dumanović et al., 2021; Sharma et al., 2022).

The enzymatic antioxidants depend on some enzymes that directly scavenge H_2_O_2_ and O_2_^•−^ and diminish their reactivity (Ahmad et al., 2010; Sharma et al., 2022) such as catalase (CAT), peroxidase (POD), polyphenol oxidase (PPO), superoxide dismutase (SOD), glutathione peroxidases (GPX), ascorbate peroxidases (APX), and glutathione reductase (GR) (Ahmad et al., 2010; Gill and Tuteja, 2010; Dumanović et al., 2021; Sharma et al., 2022). Interestingly, our greenhouse study showed that NPAAs dramatically enhanced the profile of enzymatic antioxidants of *S. sclerotiorum*-infected bean plants at 72 hpt including three antioxidant-associated enzymes CAT, POX, and PPO. It was reported previously that POX preserves redox homeostasis via regulating H_2_O_2_ levels (Rajput et al., 2021), whereas PPO is associated with the oxidation of the phenolic into reactive quinones. Moreover, findings of the current study proved that NPAAs application significantly upregulated the expression of three antioxidant-related genes including peroxisomal catalase (*PvCAT1*), copper/zinc superoxide dismutase (*PvCuZnSOD1*), and glutathione reductase (*PvGR*) in *S. sclerotiorum*-infected plants at 72 hpt. Collectively, these findings demonstrate that NPAAs considerably boost the enzymatic antioxidant defense machinery of bean plants to ease the destructive oxidative stress caused by *S. sclerotiorum*.

It was reported previously that GABA and its analog BABA prime the defense responses and enhance the abiotic stress tolerance of various plant species (Vijayakumari et al., 2016). For instance, GABA alleviates salt and drought stress in white clover (*Trifolium repens*) (Cheng et al., 2018) (Zhou et al., 2021), as well as salinity, osmotic stress in rice (*Oryza sativa*) (Sheteiwy et al., 2019). Likewise, seed priming with BABA improved the salinity stress tolerance in mung bean (*Vigna radiata*) (Jisha and Puthur, 2016a) and rice (*Oryza sativa*) seedlings (Jisha and Puthur, 2016b), as well as drought stress pepper (*Capsicum annuum*) plants (Shehu Abdulbaki et al., 2024). This might be due to the induction of myriad pre-germination mechanisms, i.e., synthesis of nucleic acids and proteins, phospholipids production, activation of DNA repair, ATP production, and antioxidant system (Saha et al., 2022).

Moreover, GABA and BABA prime defense responses against several fungal and bacterial pathogens (Li et al., 2019; Deng et al., 2020; van Rensburg and Van den Ende, 2020; Nehela and Killiny, 2023a, b; Li et al., 2024). For example, priming with GABA enhanced the response of mango (*Mangifera indica*) fruits to *Colletotrichum gloeosporioides* as well as the responses of *Arabidopsis thaliana* plants (van Rensburg and Van den Ende, 2020) and tomato (*Solanum lycopersicum*) fruits to *Botrytis cinerea* (Li et al., 2024). Similarly, GABA contributes to the defense of *A. thaliana* and sweet orange (*Citrus sinensis*) to the bacterial pathogens *Pseudomonas syringae* (Deng et al., 2020) and ‘Ca. Liberibacter Asiaticus’, respectively (Nehela and Killiny, 2023a, b). The priming effect of GABA against phytopathogens might be due to the modulation of phytohormones and their signaling pathways (Nehela and Killiny, 2023b; Li et al., 2024) and/or maintaining of redox status and buffering the antioxidant machinery (van Rensburg and Van den Ende, 2020; Nehela and Killiny, 2023a). Together, these findings suggest that NPAAs might boost the enzymatic antioxidant defense machinery as a part of the defense priming of bean plants to ease the destructive oxidative stress caused by *S. sclerotiorum*.

It was reported previously that relatively low concentrations of BABA induces resistance in several plant species against an exceptionally wide spectrum of abiotic (Jisha and Puthur, 2016a, b; Shehu Abdulbaki et al., 2024) and biotic stresses, including biotrophic and necrotrophic phytopathogens (Zimmerli et al., 2000; Ton et al., 2005; Pastor et al., 2013; Buswell et al., 2018; Luna et al., 2020). For example, *A. thaliana* plants developed full immunity against the biotrophic oomycetes *H. arabidopsidis* when treated with BABA, which acts independently from the SA- and NPR1-dependent pathway (Zimmerli et al., 2000; Ton et al., 2005), but contributed to fine-tuning of reactive oxygen species homeostasis against *P. cucumerina* (Pastor et al., 2013). Nevertheless, RBH enhanced *A. thaliana* resistance against necrotrophic *P. cucumerina* via priming of ethylene and jasmonic acid (JA) defenses (Buswell et al., 2018). Similarly, GABA improved the defense responses of tomato fruit against *B. cinerea* via modulation of ethylene and JA signaling pathways (Li et al., 2024).

On the other hand, non-enzymatic antioxidants rely on a variety of hydrophilic radical-scavenging antioxidants such as phenolics and flavonoids (Jang and Xu, 2009; Ahmad et al., 2010; Sharma et al., 2022) and lipophilic radical-scavenging antioxidants such as tocopherols and carotenoids (Lagouri, 2015). Interestingly, our greenhouse study showed that NPAAs notably enhanced the profile of non-enzymatic antioxidants of *S. sclerotiorum*-infected bean plants including total soluble phenolics and total soluble flavonoids, as well as improved the scavenging activity and antioxidant efficiency in the leaves of infected plants. Moreover, GABA and ß-alanine applications have been shown to increase the total amino acid and proline content of *S. sclerotiorum*-infected bean plants. Proline, a compatible solute, plays a pivotal role in plant stress tolerance by contributing to osmoregulation and scavenging of ROS (Ben Rejeb et al., 2014; Signorelli et al., 2014). The elevated proline content in NPAA-treated plants may contribute to their enhanced resistance to *S. sclerotiorum* infection. Together, these findings suggest that the application of NPAAs induces the non-enzymatic antioxidant defense machinery in *S. sclerotiorum*-infected plants to lessen the dangerous consequences of ROS and maintain their homeostasis.

Although current research suggests that NPAAs application might be an emerging approach in plant defense against phytopathogens, and holds potential for enhancing crop resilience, further studies are required to better understand the physio-biochemical effect of NPAAs with treated plants. Moreover, the relationship between NPAAs and other metabolic pathways, particularly phytohormones, is still poorly investigated. In the current study, we showed that seed priming combined with three root drench applications enhanced the resistance of common bean plants to *S. sclerotiorum* infection. Root applications may offer more targeted protection, especially against soil-borne pathogens, potentially complementing the systemic protection conferred by seed treatments. Future studies should investigate deeper into understanding the optimal timing, dosage, and application methods. Addressing these gaps will be crucial for unlocking the full potential of GABA application in agriculture for sustainable management practices against phytopathogens.

## 5 Conclusions

In conclusion, the experimental results suggest that GABA and *ß*-alanine have emerged as promising eco-friendly alternatives to synthetic fungicides for controlling white mold disease caused by *S. sclerotiorum* in common beans. Both NPAAs (GABA and *ß*-alanine) have demonstrated remarkable efficacy in inhibiting the growth of *S. sclerotiorum* and reducing the severity of white mold disease on common beans. Their fungistatic activity, coupled with their ability to alleviate oxidative stress, enhance antioxidant defenses, and promote plant growth, establishes them as valuable tools for sustainable bean production. Breifly, exogenous application of GABA (5.7 mM) or ß-alanine (6.08 mM) as a seed treatment followed by three root drench applications efficiently decreased the disease severity and stimulated plant growth of treated *S. sclerotiorum*-infected plants. However, further research is needed to fully understand the mechanisms underlying the antifungal activity of NPAAs, to optimize application strategies for their use in the field, and to explore their potential in integrated pest management programs. In addition, the potential environmental impacts of NPAAs should be carefully evaluated before utilization in the field. The successful implementation of GABA and *ß*-alanine as eco-friendly antagonists of *S. sclerotiorum* holds the promise of a paradigm shift in white mold disease management, fostering sustainable and environmentally sound agricultural practices.

## Data availability statement

The original contributions presented in the study are included in the article/supplementary materials. Further inquiries can be directed to the corresponding authors.

## Author contributions

YN: Conceptualization, Data curation, Formal analysis, Investigation, Methodology, Project administration, Resources, Software, Supervision, Validation, Visualization, Writing – original draft, Writing – review & editing. YM: Data curation, Funding acquisition, Resources, Software, Writing – original draft, Writing – review & editing. NG: Data curation, Formal analysis, Investigation, Methodology, Resources, Validation, Writing – original draft, Writing – review & editing. OA: Data curation, Formal analysis, Investigation, Methodology, Software, Writing – original draft, Writing – review & editing. TX: Writing – review & editing, Data curation, Formal analysis, Methodology, Resources, Software, Writing – original draft. AE: Data curation, Formal analysis, Resources, Software, Supervision, Writing – original draft, Writing – review & editing. HZ: Data curation, Formal analysis, Project administration, Resources, Supervision, Writing – original draft, Writing – review & editing. AA: Writing – original draft, Writing – review & editing, Data curation, Formal analysis, Methodology, Software. SB: Data curation, Formal analysis, Methodology, Software, Writing – original draft, Writing – review & editing. EH: Data curation, Formal analysis, Investigation, Methodology, Software, Writing – original draft, Writing – review & editing. AM: Data curation, Formal analysis, Methodology, Software, Writing – original draft, Writing – review & editing. WH: Conceptualization, Data curation, Formal analysis, Investigation, Methodology, Resources, Software, Validation, Writing – original draft, Writing – review & editing.
